# Statistics of Insanity in the United States

**Published:** 1860-07

**Authors:** Richard J. Dunglison

**Affiliations:** Physician to the Burd Orphan Asylum and to the Albion Society


ART. II.—*Statistics of Insanity in the United States.* By RICHARD J. DUNGLISON, M.D., Physician to the Burd Orphan Asylum and to the Albion Society.

STATISTICS of physical or mental infirmities interest the curious inquirer more often than they reward the zeal of the compiler. The reason is obvious; a few new facts of little consequence, or a few old ones with which he has been unfamiliar, become magnified as objects of importance in the eyes of the one, while the patience of the other receives but slight satisfaction in the paucity of useful materials separated from the confused mass of details. Matters of slight moment are dwelt upon, considered and reconsidered, while what is truly important is thrown aside, rejected as worthless, or left to the exploration of more careful observers. The latter must always, of course, in numerical strength, be the fewer, but the grand results so often obtained by patient investigation preserve the memories of their authors, when those who have devoted themselves to unimportant abstractions have been forgotten, and their results have passed away with them.

Perhaps statistics of infirmities of the organs of thesenses furnish as striking an exemplification of this fact as any other sources of statistical information. The institutions devoted to the care and comfort of the blind, deaf-mute, and insane portions of the population, do not appear equally to appreciate the value of general information upon points on which professional and unprofessional are eager to acquire knowledge. How often are we told, as if they were almost the only points which could possess any interest to us, that the institution has expended so much in the course of the year for groceries and provisions, and has laid out its grounds in such a scientific and ornamental style of gardening as to excite the admiration of all beholders! To become truly useful sources of practical information, we need, rather than an advertisement of this kind, an enumeration of all the facts bearing upon the causes of predisposition to certain morbid affections of the senses, a per-

---

sonal history of the patient's condition, sex, and age, an account of the greater prevalence of mental disease in one district of country than another, the influence of marriage, sex, and constitution, on recovery or the reverse, and the numerous other channels through which lessons may be learned to guard us from the incursion of disease, or to assist us in relieving the infirmities to which we are subject.

If we could only bring home to our minds the consciousness of the fact that we are all exposed to most of the causes, of one kind or another, which predispose or excite to blindness, deaf-dumbness, or insanity, the world at large would be much less indifferent than it now is to considerations which seem at present to possess direct interest to comparatively a small proportion. It is not easy to impress this conviction upon the mind, and perhaps it may be as well that people generally should remain indifferent to it; the gloomy contemplation would assuredly not add much of happiness to the cares of every-day life. The truth is only mentioned to show how much wider the field of accurate statistical information might become, if more general interest were excited in such subjects of universal consequence.

We are, of course, dependent upon the accuracy and careful scrutiny of others for the mass of statistics through which we must search for the elimination of but few important details. If such authorities were unanimous in their mode of arrangement, and in their knowledge of what should be accepted as useful and what might be unhesitatingly rejected as worthless, if some uniform understanding of the wants of the professional and unprofessional could be arrived at, how greatly might the whole matter of statistics be simplified! It is by no means a grateful task, while aiming at unexceptionable accuracy, to discover how much disorder has been thrown into statistical records by the total carelessness, or only near approximation to accuracy, of those who should be the most worthy sources of information. If some system of classification were adopted which would furnish facilities for the collection of facts, and be uniformly applicable to every institution in which the blind, or the deaf-mute, or the insane are cared for, the slight additional trouble of registration would be amply compensated for by the grateful appreciation of those interested in human infirmities.

Insanity, in all its phases of mental and moral perversion, is fully described in works devoted to special and general pathology. The purpose of this article is to avoid all such points as refer to symptomatology, diagnosis, or treatment, and to adhere, as far as practicable, to facts which may be confirmed or established by the numerical records and the enlarged experience of those who have devoted special attention to the subject. A complete statistical history of insanity from European and American sources is almost impracticable. Continental authorities are often inaccessible, and from a few scattering details in countries whose language is unintelligible to the rest, we can make no inferences which are worth recording. Our own country is rich in the registered history of mental ailments, and from these channels it is proposed to make such deductions and collect such materials as may furnish a condensed history of insanity in the United States. As the reports of institutions for the insane are the most prolific records it is in our power to consult, we shall collect from them statistical information upon prominent points of interest connected with the insane, having previously considered such facts, meagre as they are, as have been furnished in the details of the United States Census of 1850. We may therefore appropriately touch first upon the statistics of the insane among the general population, and afterwards upon institutions devoted to their care. We shall refer merely to those cases of mental alienation which occur from perversion of intellectual and moral qualities, and exclude entirely all reference to congenital cases of defective mental development, embraced under the head of Idiocy.

### I. STATISTICS OF THE INSANE AMONG THE GENERAL POPULATION.

Information on the number, etc. of the insane in this country can only be obtained through the details of an imperfect census. How little or how much reliance can be placed upon it, we cannot now argue; but adopting as our standard the results which it has furnished, we are able to make certain estimates of the influence of race, of the proportionate numbers of this class of unfortunates in different states, and other points of interest which may have some value to statisticians, however accurate or inaccurate the figures may be. In referring to census details we become involuntarily disbelievers in the idea that figures are infallible, and we condemn the laxity with which facts are collected, although we employ them almost as freely as if they were the most accurate results that census-takers could accomplish. The few facts obtained from the census of 1850 are embraced in the following tables.

1. *Comparison of the Insane, Blind, Deaf and Dumb, etc.*—The following table, in an aggregate of 50,994 cases of infirmities of sense or the senses, exhibits the proportion of insane to the other classes in the general population:—

| | | |
|---|---|---|
| Insane . . . . . | 15,610, or 1 to 1485 | of general population. |
| Idiots . . . . | 15,787, or 1 to 1469 | " " |
| Deaf-mutes . . . . | 9,803, or 1 to 2365 | " " |
| Blind . . . . | 9,794, or 1 to 2367 | " " |

The blind approximate closely the number of the deaf and dumb, while the insane and idiotic classes are not widely apart from each other. The insane are more than 60 per cent. more numerous, if we can place reliance on these estimates, than either the blind or deaf-mutes.

*In every* 100,000 *of the Population* (*Census* 1850) *there are:—*

| | |
|---|---|
| Insane . . . . . . 67. | Blind . . . . . . . 42. |
| Idiots . . . . . . . 68. | Deaf-mutes . . . . 42. |

2. In a previous article upon deaf-muteism,* we referred to the greater prevalence of that infirmity among the native than among the foreign population. If we adopt a similar mode of classification with the insane, excluding slaves only, we shall find insanity far more prevalent among the foreign-born than among the native population.

PROPORTION OF INSANE IN THE NATIVE AND FOREIGN POPULATION.

| STATES. | Native Insane. | Native Population. | Proportion to Pop. | Foreign Insane. | Foreign Population. | Proportion to Pop. |
|---|---|---|---|---|---|---|
| New England........ | 3,375 | 2,423,223 | 1 : 718 | 412 | 299,340 | 1 : 726 |
| New York, New Jersey, and Pennsylvania............ | 3,667 | 4,884,356 | 1 : 1332 | 1,029 | 1,005,036 | 1 : 976 |
| N. W. States and Territories......... | 1,963 | 4,656,158 | 1 : 2372 | 340 | 638,784 | 1 : 1878 |
| Slave States, etc.... | 3,860 | 5,534,813 | 1 : 1434 | 268 | 232,839 | 1 : 870 |
| | 12,865 | 17,498,550 | 1 : 1360 | 2,049 | 2,175,999 | 1 : 1061 |

Change of mode of living, intemperance and dissipation, the frustration of fondly-indulged hopes of success, reverses of fortune, etc., are very often followed by insanity, and these, we imagine, operate powerfully among the foreign-born population. In the words of one of the New England Institutions: "Driven from their early homes by poverty, ignorance, and delusive hopes, they are thrown on our shores, and left to contend as they may with the new circumstances around them, until disappointment or sickness, or intemperance, or other form of vice, extinguishes the feeble light of reason, and consigns them to a lunatic hospital. They are unpromising patients. They do not recover in so large a proportion as others, and consequently contribute largely to swell the number of incurable cases which crowd the wards of our hospitals."†

We do not offer any explanation why mental alienation should attack the foreign-born in so large a proportion in some States, nor why they should be attacked in a proportion in the New England States no greater than exists among the native born. Reasons doubtless exist, which must be apparent to those familiar with the intimate domestic arrangements, habits, and feelings of each class.

* "Observations on the Deaf and Dumb," reprinted from the N. A. Medico-Chirurgical Review, Philadelphia, 1858.

† Third Annual Report of the State Lunatic Hospital at Northampton, Mass., 1858, p. 31.

3. *Influence of Race.*—It will be seen, by the following table, that insanity is far more prevalent among the white and free colored population of the United States than among the slaves. It would be natural to suppose that absence of care and freedom from anxiety as to the future would tranquilize the mind, and ward off disturbing elements. The condition of a happy slave, thinking only of the present, and not dreaming of want in the future, would appear to be that which should give rise very infrequently to causes of insanity. Intemperance, so fruitful a source of misery and unhappiness to the white and free colored, is comparatively unknown among the slave population, every slaveholder striving to prohibit the use of alcoholic liquors, and dreading the proximity of taverns or drinking-places, which can supply unlimited indulgence to his slave. Hence, intemperance itself does not exist as a cause of mental alienation, nor can the thousand evils it always brings in its train—disease, exposure, excesses, etc.—be worthy of consideration as causes of insanity. Anxiety would be a far more productive source of mental disease to the free colored population, from the fact that they have to contend at a disadvantage with the whites in many of their occupations and modes of earning a livelihood. It would be interesting to pursue these inquiries further than they have already been carried, to determine what causes are mainly at work to produce insanity among the colored race, and why in some portions of the country—New England and Delaware for example—the proportion should be so large. It may be remarked, however, that in a small population, in Maine, for instance, the number of insane must be so small that no very reliable deductions can be founded upon such meagre statistics. Yet New England, which contains but about one-thirtieth of the free colored population of the Union, had within her limits nearly an eighth of the free colored insane. Ohio, with a larger free colored population than that of all the New England States, had, in 1850, but one-third as many insane; while Maryland and Virginia had but two and a half times as large an insane free colored population as New England, although having more than five times the number of free colored in the general population.

In inserting the following table, which has been carefully prepared from the census of 1850, we desire to correct an erroneous conclusion which has crept into a valuable statistical work—*Boudin's Traité de Géographie et de Statistique Médicales, etc.*, Paris, 1857 (vol. ii. p. 143)—in consequence of figures adduced by Dr. Nott, of Mobile, to show that the farther removed the negro may be from the tropics, the more liable will he become to mental alienation.* M. Boudin asks: "Of what consequence is it, if the

* J. Nott, M.D. Two Lectures on the Natural History of the Caucasian and Negro Races. Mobile, 1844.

negro is able to succeed in living and even of perpetuating his race in a temperate zone, if it should be shown, as has been attempted, that he becomes insane there in an enormous proportion?" A reference to the table disproves this assertion, and answers M. Boudin's questions satisfactorily. The farther north we go, the less numerous do the slaves become, until at last we find all the colored race free. It is then that causes of insanity become multiplied to them, while climate, which is not a powerful cause of perversion of the mental and moral faculties, would operate more actively as a predisposing cause of idiocy.

*Influence of Race in the production of Insanity.*
(CENSUS OF 1850.)

| | STATES. | White Insane. | Proportion to White Population. | Free Col. Insane. | Proportion to Free Col. Population. | Slave Insane. | Proportion to Slave Population. |
|---|---|---|---|---|---|---|---|
| 6 New England States. | Maine . . . . | 556 | 1 : 1046 | 5 | 1 : 271 | | |
| | New Hampshire . | 378 | 1 : 839 | | | | |
| | Vermont . . . | 560 | 1 : 560 | | | | |
| | Massachusetts . | 1661 | 1 : 593 | 19 | 1 : 477 | | |
| | Rhode Island . . | 210 | 1 : 685 | 7 | 1 : 524 | | |
| | Connecticut . . | 464 | 1 : 782 | 6 | 1 : 1282 | | |
| 3 Middle States. | New York . . . | 2487 | 1 : 1225 | 34 | 1 : 1443 | | |
| | New Jersey. . . | 370 | 1 : 1258 | 9 | 1 : 2634 | | |
| | Pennsylvania . . | 1865 | 1 : 1210 | 49 | 1 : 1094 | | |
| 15 Slave States and Dis. of Columb. | Delaware . . . | 48 | 1 : 1482 | 20 | 1 : 903 | | |
| | Maryland . . . | 477 | 1 : 876 | 44 | 1 : 1698 | 25 | 1 : 3,614 |
| | Virginia . . . | 864 | 1 : 1035 | 47 | 1 : 1156 | 59 | 1 : 8,008 |
| | North Carolina . | 467 | 1 : 1184 | 10 | 1 : 2746 | 33 | 1 : 8,743 |
| | South Carolina . | 224 | 1 : 1225 | 4 | 1 : 2240 | 21 | 1 : 18,330 |
| | Georgia . . . . | 294 | 1 : 1774 | 2 | 1 : 1465 | 28 | 1 : 13,631 |
| | Alabama . . . | 201 | 1 : 2122 | 2 | 1 : 1132 | 30 | 1 : 11,428 |
| | Florida . . . . | 9 | 1 : 5245 | | | 2 | 1 : 19,655 |
| | Mississippi. . . | 105 | 1 : 2816 | | | 24 | 1 : 12,911 |
| | Louisiana . . . | 144 | 1 : 1774 | 11 | 1 : 1587 | 45 | 1 : 5,440 |
| | Texas . . . . | 37 | 1 : 4163 | | | | |
| | Arkansas . . . | 60 | 1 : 2703 | | | 3 | 1 : 15,700 |
| | Missouri . . . | 249 | 1 : 2377 | 2 | 1 : 1309 | 11 | 1 : 7,947 |
| | Kentucky . . . | 502 | 1 : 1516 | 2 | 1 : 5005 | 23 | 1 : 9,173 |
| | Tennessee . . . | 380 | 1 : 1991 | 5 | 1 : 1284 | 22 | 1 : 10,884 |
| | Dist. of Columbia. | 13 | 1 : 2918 | 9 | 1 : 1117 | 1 | 1 : 3,687 |
| 7 West'n States and 4 Territories. | Ohio . . . . . | 1303 | 1 : 1500 | 14 | 1 : 1805 | | |
| | Indiana . . . . | 556 | 1 : 1757 | 7 | 1 : 1609 | | |
| | Illinois . . . . | 236 | 1 : 3584 | 2 | 1 : 2718 | | |
| | Michigan . . . | 132 | 1 : 2992 | 1 | 1 : 2583 | | |
| | Iowa . . . . . | 42 | 1 : 4568 | | | | |
| | Wisconsin . . . | 54 | 1 : 5643 | | | | |
| | California . . . | 2 | 1 : 45817 | | | | |
| | Minnesota, New Mexico, Utah, and Oregon . . | 22 | 1 : 4181 | | | | |
| | Total. . . . . | 14,972 | 1 : 1305 | 311 | 1 : 1397 | 327 | 1 : 9799 |

4. The question may arise, *Has insanity increased in the United States in a greater ratio than the population?* Discrepancies certainly exist between the censuses of 1840 and 1850 sufficient to warrant us in forming one of two hypotheses: either insanity has been on the increase or the returns of the census upon this point are unreliable. We are more disposed to assume the latter position, for reasons which have already been stated. In the census of 1840 the idiotic and insane were not separated in the statistics, while in 1850 an independent enumeration of each class was made. We cannot more appropriately refer to questions arising out of the comparison of the two censuses, than by citing the remarks of Prof. George Tucker* on the results obtained in each: "By this comparison it appears that of the insane and idiotic—

| | | | |
|---|---|---|---|
| In the white population, the proportion in | 1840 | was as | 1 in 977 |
| " " " " " | 1850 | " | 1 in 688 |
| In the colored " " " | 1840 | " | 1 in 978 |
| " " " " " | 1850 | " | 1 in 1929 |

The suspicions entertained against the accuracy of that part of the census of 1840 which respected the insane of the colored population, have been justified by subsequent investigations; but, on the other hand, in correcting the error, the correspondent part of the seventh census (1850) seems hardly entitled to our entire confidence. We know that much sensibility was excited by the greater frequency of insanity among the colored race which resulted from that census, and it is possible that the interest thus felt may, in more ways than one, have biased the judgment of the census-takers in placing individuals under this class. Though the census of 1840 unquestionably overrated the number of the colored insane in the Northern States, yet when we saw the proportion gradually increase as we proceeded on the Atlantic coast from Georgia to Maine, and in the West from Louisiana to Michigan, it was not to be believed that the diversity was produced by a correspondent variety and gradation of errors; and, reasoning on probabilities, we were compelled to admit that there was some solid foundation for the difference exhibited, though it might be greatly exaggerated. We may add, that there is intrinsic evidence in favor of the census of 1840 on this point, which that of 1850 does not possess. Nor is this all. That census itself affords grounds for questioning its accuracy. It shows that while in the white population the proportion of the insane and idiotic is as much as 1 in 668, in the colored population it is only 1 in 1929; and though we cannot admit

* Progress of the United States in Population and Wealth in fifty years. Appendix, p. 24. 1856.

that, in New England, where the colored population shows a small increase, the number of insane and idiotic has fallen from 383 to 45 [55 ?], as the census shows; neither can we readily believe that, contrary to all previous enumerations, the proportion of the white race thus afflicted is three times as great as that of the colored. We must, then, look to future enumerations to decide whether the liability of the last-mentioned race to these mental maladies, which the census of 1840 has confessedly exaggerated in some States, has not been generally underrated by the census of 1850, and whether truth does not occupy a middle point between them." We cannot, therefore, decide whether insanity has increased generally, or whether the influence of race has been exhibited in either an increase or diminution of the tendency to mental alienation in the white or the colored portions of our population.

### II. STATISTICS OF THE INSANE, DERIVED FROM INSANE INSTITUTIONS.

The annals of the rise and progress of United States Institutions for the Insane may be briefly told. It is the history of philanthropic efforts to protect and relieve a numerous class of unfortunates, to shelter them from injurious influences, and to afford them a home, where, under the attentive care of those who would take an active interest in them, they might be placed under the best possible conditions for improvement or recovery. We cannot furnish a condensed historical account more appropriately than by adopting the brief statement which has already been published by one who has himself had such abundant opportunities of seeing

"That noble and most sovereign reason,
Like sweet bells jangled, out of tune and harsh."

### 1. HISTORICAL SKETCH OF INSTITUTIONS FOR THE INSANE IN THE UNITED STATES.*

"If we take a retrospective glance over a period of less than half a century, we find that, in 1815, throughout the whole domain of the United States, the only separate independent public institution for the insane, was that at Williamsburg, Virginia. That establishment had undergone serious vicissitudes. It was opened during our colonial dependence upon Great Britain, and, in the course of the Revolution, its operations were suspended, and the buildings converted into military barracks.

"In the latter half of the decennium ending on the 31st of December, 1820, two new institutions were opened. These were, the Asylum at Frankford, now

* Dr. Pliny Earle, "Reports of American Institutions for the Insane," American Journal of the Medical Sciences, April 1857, pp. 442–3.

Philadelphia, in 1817; and the McLean Asylum, in what is now Somerville, Massachusetts, in 1818.

"In the course of the decennium terminating with the close of 1830, five establishments for the insane sprang into existence. The Bloomingdale Asylum went into operation in 1821; the Retreat at Hartford, Connecticut, and the Asylum at Lexington, Kentucky, in 1824; and the Asylums at Staunton, Virginia, and Columbia, South Carolina, in 1828.

"Earliest in the next succeeding period of ten years was the Massachusetts State Hospital, at Worcester, which was opened in 1833; the Vermont State Asylum, at Brattleboro', followed in 1836; the Ohio State Asylum, at Columbus, in 1838; the pauper institutions for the cities of Boston and New York, in 1839; and the Maine State Asylum, at Augusta, in 1840. It was during this period that the greatest impulse was given to the scheme for meliorating the condition of the insane in these United States. In the production of this impulse, no person exerted greater influence than the late Dr. Samuel B. Woodward, who was at that time Superintendent of the Hospital at Worcester. The zeal and hopefulness with which he ever pursued his occupation; the moral glow of sunlight which he disseminated all around him, over a sphere thitherto almost universally regarded, in the popular mind, as shrouded with clouds and involved in darkness; and the elaborate reports which, emanating from his pen, were scattered broadly throughout the country, all contributed to awaken an interest in the subject which had never been previously manifested.

"The decade from 1840 to 1850 exhibited the effect of this increased interest. The Pennsylvania Hospital for the Insane was opened in 1841. In 1841 or 1842, a separate building was erected for the pauper insane of King's County, New York. The New Hampshire State Asylum, at Concord, and the Mt. Hope Institution, at Baltimore, commenced operations in 1842; the Asylum at Utica, New York, in 1843; the Butler Hospital, at Providence, R. I., in 1847; and the State Asylums at Trenton, N. J., Indianapolis, Ind., and Jackson, Louisiana, in 1848. About the middle of this decade, the Maryland Hospital, at Baltimore, theretofore devoted to the treatment of general diseases, was converted into an institution exclusively for the insane. No positive information upon the subject is now accessible to us; but it is our impression, relying upon memory, that the original Asylum at Nashville, Tennessee, and the Asylum at Milledgeville, Georgia, were opened in the early part of this decennium.

"Since 1850, several institutions have been brought into existence. The State Hospitals, at Harrisburg, Pa., Fulton, Missouri, and Jacksonville, Illinois, were organized and first received patients in 1851. The new building of the Tennessee Hospital was so far completed as to be occupied in 1852. The State Asylum at Stockton, California, and the Asylum for the County of Hamilton, Ohio, were opened in 1853; the State Institutions at Taunton, Mass., and Hopkinsville, Kentucky, in 1854; the United States Government Hospital, near Washington; the State Asylum at Jackson, Mississippi, and those at Newburg and Dayton, Ohio, in 1855; and the State Asylum at Raleigh, N. C., in 1856. The new building for the pauper insane of King's County, N. Y., was first occupied by patients in 1855.

"We know of but five private establishments for the treatment of the insane in the United States. The oldest of these is that of Dr. Cutter, at Pepperell, Massachusetts. The late Dr. James Macdonald established a private institute at Murray Hill, New York City, about the year 1837, and some years afterwards removed it to Flushing, Long Island, where it is still continued under the title of Sanford Hall, and in charge of the doctor's brother, General Allan Macdonald. It is one of the most complete and beautiful establishments of the kind in the world. Dr. Edward Jarvis has an establishment at Dorchester, Massachusetts; Dr. Edward Mead one near Cincinnati, Ohio; and Dr. George Cook one at Canandaigua, New York."

Since that time, others have been founded; their names and their dates of opening are furnished in the "The Table of Institutions for the Insane in the United States," on the next page. Their rapid increase in the last twenty years evinces a lively interest on the part of the people of the different States in one of the most numerous classes of infirmities.

The table has been carefully prepared, with a view of affording an opportunity of learning how much has been done in this country to ameliorate the condition of those suffering from mental alienation. Every institution in the United States, whose statistics were otherwise imperfectly obtainable, except that in California, has been applied to for information. Most of them have responded to our desire for accuracy, and we regret that the silence of others may, in a few instances, prevent us from making the record complete. Some of the institutions are supported by appropriations from State Legislatures, some are incorporated, others are pauper institutions connected with the system of city government. Insane inmates may be found in every almshouse, but it has not been deemed expedient to give such an arrangement any prominence, unless the portion devoted to the insane is a separate department under the charge of a superintendent or physician. The word "mixed" implies that the establishment is incorporated but has received aid from the State. The recoveries, deaths, etc., are not furnished as comparative tables of the results of treatment of different institutions, so widely scattered, and therefore so variously exposed to favorable or unfavorable circumstances of climate or locality, but as a sufficiently satisfactory method of condensing the history of each institution, and of exhibiting the number of insane who have received relief from their mental sufferings by recovery or death. Where no mention is made of the time from which the admissions are calculated, it may be inferred that the statistics date back to the opening of the institution.

TABLE OF INSTITUTIONS FOR THE

| | State. | Location. | Name of Institution. | Foundation. | Date of Opening. |
|---|---|---|---|---|---|
| 1 | Maine | Augusta. | Maine Insane Hospital. | State Inst. | Oct. 14, 1840. |
| 2 | New Hampshire.. | Concord. | N. H. Asylum for the Insane. | " | Oct. 29, 1842. |
| 3 | Vermont | Brattleboro'. | Vermont Asylum for the Insane. | " | Dec. 12, 1836. |
| 4 | Massachusetts | Worcester. | Mass. State Lunatic Hospital. | " | Jan. 18, 1833. |
| 5 | " | Taunton. | " " " | " | April 7, 1854. |
| 6 | " | Northampton. | " " " | " | Aug. 16, 1858. |
| 7 | " | Somerville. | McLean Asylum for the Insane. | Corporate Inst. | Oct. 6, 1818. |
| 8 | " | South Boston. | Boston City Lunatic Asylum. | Pauper " | ................... |
| 9 | " | Pepperell. | | Private " | ................... |
| 10 | " | Dorchester. | Dr. Jarvis's Private Asylum. | " " | 1843. |
| 11 | Rhode Island | Providence. | Butler Hospital for the Insane. | Corporate " | Dec. 1, 1847. |
| 12 | Connecticut | Hartford. | Retreat for the Insane. | " " | April 1, 1824. |
| 13 | " | Litchfield. | ........................................... | Private " | Oct. 1858. |
| 14 | New York | Utica. | New York State Lunatic Asylum. | State " | Jan. 16, 1843. |
| 15 | " | Blackwell's I., N. Y. | New York City Lunatic Asylum. | Pauper " | June 10, 1839. |
| 16 | " | New York City. | Bloomingdale Asylum for the Insane. | Corporate " | June 10, 1821. |
| 17 | " | Flatbush, L. I. | King's Co. Lunatic Asylum. | Pauper " | Nov. 1, 1855. |
| 18 | " | Flushing. | Sanford Hall. | Private " | May, 1846. |
| 19 | " | Canandaigua. | Brigham Hall. | Private " | Oct. 3, 1855. |
| 20 | " | Auburn. | N. Y. State Lun. As. for In. Conv. | State " | Nov. 1858. |
| 21 | New Jersey | Trenton. | New Jersey State Lunatic Asylum. | " " | May 15, 1848. |
| 22 | Pennsylvania | Philadelphia. | Penn'a Hospital for the Insane. | Corporate " | Jan. 1, 1841. |
| 23 | " | Harrisburg. | State Lunatic Hospital of Penn'a. | State " | Oct. 1, 1851. |
| 24 | " | Near Pittsburg. | Western Pennsylvania Hospital for the Insane. | Mixed " | March 13, 1856. |
| 25 | " | Frankford, (Phil'a.) | Asylum for the Relief of Persons deprived of the use of their reason | Corporate " | May 15, 1817. |
| 26 | " | Philadelphia. | Insane Department of the Philadelphia Hospital. | Pauper " | ................... |
| 27 | " | Delaware Co. | Clifton Hall. | Private " | 1860. |
| 28 | Maryland | Baltimore. | Maryland Hospital for the Insane. | State " | Jan. 1, 1834. |
| 29 | " | Near Baltimore. | Mt. Hope Institution. | Mixed " | 1842. |
| 30 | Dis. of Columbia. | Near Washington. | U. S. Government Hospital for the Insane. | U. S. " | Jan. 15, 1855. |
| 31 | Virginia | Staunton. | Western Lunatic Asylum of Va. | State Inst. | 1828. |
| 32 | " | Williamsburg. | Eastern " " | " | |
| 33 | North Carolina... | Raleigh. | Insane Asylum of North Carolina. | " | Feb. 22, 1856. |
| 34 | South Carolina... | Columbia. | State Lunatic Asylum of S. Ca. | " | Jan. 1, 1828. |
| 35 | Georgia | Milledgeville. | ........................................... | " | ................... |
| 36 | Mississippi | Jackson. | Mississippi State Lunatic Asylum. | " | Jan. 8, 1855. |
| 37 | Louisiana | Jackson. | Insane Asylum of the State of La. | " | Nov. 21, 1848. |
| 38 | Texas | Austin. | Texas State Lunatic Hospital. | " | ................... |
| 39 | Missouri | Fulton. | State Lunatic Asylum of Mo. | " | Dec. 1, 1851. |
| 40 | Kentucky | Lexington. | Kentucky Eastern Lunatic Asylum | " | 1824. |
| 41 | " | Hopkinsville. | Western Lunatic Asylum of the State of Kentucky. | " | Sept. 18, 1854. |
| 42 | Tennessee | Near Nashville. | Tennessee Hospital for the Insane. | " | March 1, 1852. |
| 43 | Ohio | Columbus. | Central Ohio Lunatic Asylum. | " | Nov. 30, 1838. |
| 44 | " | Newburg. | Northern " " | " | March 5, 1855. |
| 45 | " | Dayton. | Southern " " | " | Sept. 1, 1855. |
| 46 | " | Mill Creek. | Hamilton Co. Lunatic Asylum. | Pauper Inst. | Nov. 7, 1853. |
| 47 | " | Near Cincinnati. | Retreat for the Insane. | Private " | ................... |
| 48 | Indiana | Indianapolis. | Indiana Hospital for the Insane. | State Inst. | Nov. 21, 1848. |
| 49 | Illinois | Jacksonville. | Illinois State Hospital for the Insane. | " | Nov. 3, 1851. |
| 50 | Michigan (*h*) | Kalamazoo. | ........................................... | " | ................... |
| 51 | California | Stockton. | State Insane Asylum of California. | " | May 14, 1852. |

(*a*) The Institution commenced operations with the reception of 228 cases, principally chronic, 213 being from the other State institutions.

(*b*) Most of those admitted were females.

(*c*) Statistics from 1847 to 1859 only given.

(*d*) Ceased to receive State and County paupers in 1849.

(*e*) The average number in the Institution is here given.

(*f*) Statistics date from April 1, 1856.

(*g*) Statistics date from July 1, 1836.

(*h*) Its opening delayed by a disastrous fire.

INSANE IN THE UNITED STATES.

| Admissions. | Recoveries. | Deaths. | Remaining at last accounts. | | | Date of Statistics. | Name of Superintendent or Physician. |
|---|---|---|---|---|---|---|---|
| | | | Males. | Females. | Total. | | |
| 2127 | 871 | 267 | 129 | 108 | 237 | Nov. 30, 1859. | Dr. Henry M. Harlow. |
| 1650 | 727 | 172 | 94 | 88 | 182 | May 31, 1859. | Dr. Jesse P. Bancroft. |
| 3025 | 1433 | | 212 | 219 | 431 | Aug. 1, 1859. | Dr. W. H. Rockwell, |
| 5996 | 2747 | 696 | 152 | 165 | 317 | Sept. 30, 1859. | Dr. Merrick Bemis. |
| 1343 | 432 | 227 | 165 | 176 | 341 | Sept. 30, 1859. | Dr. George C. S. Choate. |
| 321 | Not given (*a*) | 19 | 98 | 135 | 233 | Sept. 30, 1859. | Dr. Wm. Henry Prince. |
| 4582 | 2130 | 546 | 85 | 90 | 175 | Jan. 1, 1860. | Dr. John E. Tyler. |
| ........ | ........ | ........ | ........ | ........ | ........ | ........ | Dr. Clement A. Walker. |
| ........ | ........ | ........ | ........ | ........ | ........ | ........ | |
| About 100 (*b*) | ........ | ........ | ........ | ........ | ........ | March 5, 1860. | Dr. Edward Jarvis. |
| 904 | 296 | 180 | 68 | 67 | 135 | Dec. 31, 1859. | Dr. Isaac Ray. |
| 3407 | 1643 | 347 | 105 | 110 | 215 | March 31, 1859. | Dr. John S. Butler. |
| ........ | ........ | ........ | ........ | ........ | ........ | June 16, 1860. | Dr. Henry W. Buel. |
| 5828 | 2340 | 671 | ........ | ........ | 519 | Dec. 1, 1859. | Dr. John P. Gray. |
| 5836 | 2569 | 1492 | 287 | 424 | 711 | Dec. 31, 1859. (*c*) | Dr. M. H. Ranney. |
| 1923 | 802 | 274 | 68 | 84 | 152 | Dec. 31, 1859. (*d*) | Dr. D. Tilden Brown. |
| ........ | ........ | ........ | 122 | 168 | 290 | July 31, 1859. | Dr. Edward R. Chapin. |
| ........ | ........ | ........ | ........ | ........ | 34 (*e*) | March 9, 1860. | Dr. Benj. Ogden, Visiting Phy. |
| | | | | | | | Dr. J. W. Barstow, Resident Phy. |
| 139 | 42 | 10 | ........ | ........ | 40 | Feb. 18, 1860. | Drs. George Cook and John B. Chapin. |
| 55 | 2 | | | | 51 | Sept. 1859. | Dr. Edward Hall. |
| 1563 | 605 | 206 | 141 | 165 | 306 | Dec. 31, 1859. | Dr. H. A. Buttolph. |
| 3360 | 1656 | 363 | 147 | 122 | 269 | May 25, 1860. | Dr. Thomas S. Kirkbride. |
| 1192 | 205 | 170 | 149 | 125 | 274 | Dec. 31, 1859. | Dr. John Curwen. |
| 332 | 119 | 33 | 61 | 39 | 100 | Jan. 1, 1860. (*f*) | Dr. Joseph A. Reed. |
| 1412 | 636 | 195 | 29 | 29 | 58 | March 1, 1860. | Dr. Joshua H. Worthington. |
| ........ | ........ | ........ | ........ | ........ | ........ | ........ | Dr. S. W. Butler |
| ........ | ........ | ........ | ........ | ........ | ........ | March 1, 1860. | Dr. Robert A. Given. |
| 1615 | 664 | 231 | 54 | 52 | 106 | Dec. 31, 1859. | Dr. John Fonerden. |
| 1963 | ........ | ........ | 71 | 106 | 177 | Jan. 1, 1860. | Dr. Wm. H. Stokes. |
| 270 | 53 | 47 | ........ | ........ | 138 | July 1, 1859. | Dr. Charles H. Nichols. |
| 1576 | 640 | 381 | 219 | 153 | 372 | Oct. 1, 1859. (*g*) | Dr. Francis T. Stribling. |
| ........ | ........ | ........ | 147 | 110 | 257 | Oct. 1, 1857. | Dr. John M. Galt. |
| 233 | 41 | 18 | 95 | 52 | 147 | Nov. 1, 1858. | Dr. Edward C. Fisher. |
| 1139 | 468 | 251 | 99 | 95 | 194 | Nov. 5, 1859. | Dr. J. W. Parker. |
| ........ | ........ | ........ | ........ | ........ | ........ | ........ | Dr. Thomas F. Green. |
| 260 | 65 | 30 | 65 | 41 | 106 | Oct. 1, 1859. | Dr. Robert Kells. |
| 851 | 225 | 374 | 89 | 68 | 157 | Dec. 31, 1859. | Dr. J. D. Barkdull. |
| ........ | ........ | ........ | ........ | ........ | ........ | Nov. 27, 1857. | Dr. J. C. Perry. |
| 426 | 133 | 79 | 97 | 74 | 171 | Nov. 24, 1858. | Dr. T. R. H. Smith. |
| 2389 | 882 | 926 | 130 | 98 | 228 | Sept. 30, 1859. | Dr. W. S. Chipley. |
| 443 | 111 | 91 | 107 | 97 | 204 | Dec. 1, 1859. | Dr. Francis G. Montgomery. |
| 390 | 109 | 45 | 96 | 62 | 158 | Oct. 1, 1857. | Dr. W. A. Cheatham. |
| 3480 | 1792 | 441 | 111 | 103 | 214 | Nov. 1, 1859 | Dr. R. Hills. |
| 426 | 179 | 19 | ........ | ........ | 148 | Oct. 31, 1857. | Dr. R. C. Hopkins. |
| 502 | ........ | 40 | 81 | 79 | 160 | Nov. 1, 1858. | Dr. J. J. McIlhenny. |
| 913 | 246 | 87 | 143 | 130 | 273 | June 5, 1859. | Dr. Wm. Mount. |
| ........ | ........ | ........ | ........ | ........ | ........ | ........ | Dr. Edward Mead. |
| 1753 | 819 | 128 | 151 | 152 | 303 | Oct. 1, 1859. | Dr. James S. Athon. |
| 1018 | 430 | 92 | ........ | ........ | 229 | Dec. 1, 1858. | Dr. Andrew McFarland. |
| ........ | ........ | ........ | ........ | ........ | ........ | ........ | Dr. E. H. Van Deusen. |
| 865 | 480 | 61 | ........ | ........ | 162 | Dec. 31, 1855. | Dr. W. D. Aylett. |

NOTE.—One or two changes may have occurred from death or resignation among the superintendents above mentioned, but we have given, as far as was in our power, those whose names appeared upon the latest reports. To those who kindly responded by letter or transmitted to us their reports in answer to our application for information, we desire to return our sincere thanks; and especially do we feel grateful to Dr. Kirkbride and Dr. Edward A. Smith, of the Pennsylvania Hospital for the Insane, for facilities extended to us in our endeavors to furnish accurate and reliable statistics.

A sketch has thus been briefly given of the means which have been afforded by humane sympathy for the care and protection of the insane. That the power of some States to furnish adequate comfort to them is limited, is to be greatly regretted; yet many of the younger institutions are still in their infancy, and have not attained their full development. As they progress in their usefulness, and as soon as the public realize by more accurate statistical details how numerous the unfortunate insane are, in proportion to the number accommodated in insane institutions, fewer patients will remain at home to be a source of anxiety to their families, and the comforts of a home in which they can receive constant medical attention, and be removed from the causes of their malady, will be more fully appreciated.

Facts collected from the materials furnished by so many institutions would, if carefully tabulated, supply statistical information of importance to those of the medical profession who are interested in them. The scanty details from some of the institutions assist in making up a total, but the care with which some of the others have examined into facts connected with the insane, and collated them for the benefit of the public, deserves especial commendation. So many points arise, however, in the consideration of such infirmities, which are of more interest to those connected with institutions than to the profession, that it is proposed to limit the field of our inquiries to questions of general importance which do not exclusively belong to the special province of institutions for the insane only.

Some of the points may be studied as part of the personal history of the patient previous to the attack of insanity, such as the age, sex, conjugal condition, occupation, etc., while others belong more properly to the history of the attack, such as the period of the year, the particular form which the disease may assume, the cause of death, etc. If the attack should not be the first from which he had suffered, we should naturally refer to the previous history of the disease, and learn something of the duration and number of former attacks. Therapeutically, we shall have nothing to say here; to discuss merely a few pathological and psychological points of interest which seem to accumulate upon us at every step, we feel to be a task promising to claim our utmost capacity of time and space. Several considerations would arise, that cannot be properly placed under any of the heads we have mentioned, such as the general liability to second attacks, etc. These, though perhaps indirectly connected with the history of the attack, may be arranged with more advantage under the history of the recurrence of insanity. Adopting the mode of classification of subjects above given, we shall refer first to

2. THE PERSONAL HISTORY OF THE PATIENT PREVIOUS TO THE ATTACK.

This is abundantly illustrated in the statistics of almost all our institutions.

*a. Sex.*—Of the numerous circumstances under which the female sex is placed by her physical nature, her temperament and sympathies, we might infer that many are favorable to the production of mental disorders, and that woman would, on this account, be more liable to insanity than the other sex. It has been truly said by Cabanis,* that "by a severe necessity attached to the rôle which nature assigns to her, woman is subjected to many accidents and inconveniences; her life is nearly always a series of alternations of health and suffering; and too often the suffering predominates." Yet her constitution resists shocks and trials under which the more robust nature of man would shrink, and her delicate organization seems often to acquire strength after one series of trials to be prepared for those which are to follow.

In viewing the relation of the sexes, in the liability to mental aberration, we must of course bear in mind the greater proportion of males or females in the general population. Erroneous conclusions have in many cases been drawn from the mere fact of the number of insane females being greater in certain portions of the country, to found a theory that the male sex is less liable to insanity than the female. May not this deviation be owing, in some measure, to a difference of occupation or of the circumstances predisposing to mental disorder in the males or females of one part of the United States from those of another? For even if the number of insane females should preserve the same general ratio to the female population, the devotion of the other sex to a different class of occupations might alter the ratios very sensibly. It has been exhibited in the experience of several transatlantic observers, that psychical affections are far more prevalent in manufacturing than in agricultural districts—a fact which might perhaps affect the male ratio of the insane more than the female. The mortality has also been found by statistics to be greater in the male sex than in the female, being estimated by one authority at 50 per cent. greater; while the female recovers more frequently from insanity than the male. We have to distinguish, therefore, between the number of either sex who have actually become insane and those who are found to be so, when mortality or recovery has exerted an influence to modify the ratio. And besides this, moral causes, such as grief, anxiety, etc., affect women more than men, and the chances of recovery being greater in moral causes than in physical, of course fewer women remain for any length of time insane.

* Rapport du Physique et du Moral de l' Homme, tome i. p. 350. Paris, 1802.

The following details have been collected from the experience of more than forty of the institutions of this country, either during the last year of their recorded observations or throughout the whole course of their career.

*Sex of the Insane in United States Institutions.*

| | Males. | Females. | Total. |
|---|---|---|---|
| U. S. Gov't and State Institutions . | 15,328 | 13,232 | 28,560 |
| Corporate Institutions . . . . . | 6,245 | 5,793 | 12,038 |
| Mixed " . . . . . | 597 | 497 | 1,094 |
| Pauper " . . . . . | 3,423 | 3,880 | 7,303 |
| Total . . . . . . | 25,593 | 23,402 | 48,995 |

In June, 1850, Dr. Edward Jarvis, of Dorchester, Massachusetts, presented to the Association of Medical Superintendents of American Institutions for the Insane, a paper on the "Comparative Liability of Males and Females to Insanity, and their Comparative Curability and Mortality when Insane." His statistics of twenty-one American hospitals represent the proportion of males insane to females as 121 to 100 in a total of 24,573 cases. Our own estimate is founded upon nearly 25,000 more cases than are embraced in the investigations of Dr. Jarvis, and we find a much less difference in the proportion of the sexes than that determined upon at that time: 100 females are found by our own estimates to be insane—judging merely from hospital records—to every 109 males. But the sexes are not represented in the institutions as they are in the general population. We shall hereafter find that the period of life embraced between the years of 20 and 50 is that in which mental disorder is of much more frequent occurrence than after or before those ages. We must, therefore, in order to learn what ratio the sexes bear to each other on the question of insanity between the ages of 20 and 50, determine also their relative number in the general population. The census of 1850, which we may—with some hesitation, perhaps—take as a convenient guide, gives a proportion of 108 males to 100 females in the general population between the ages of 20 and 50. The number corresponds closely enough with that given in the general ratio of the sexes in insane institutions; but then we have to assume that those figures not only correspond with the proportion outside of the institution, but also that, taken without regard to age, they represent the proportion of the sexes under 20 and over 50 years of age—two difficulties which we do not attempt further to solve.

Dr. Thurnham,* in examining this question, in reference especially to

* On the "Relative Liability of the Two Sexes to Insanity." Quarterly Journal of the Statistical Society of London, December, 1844.

European institutions, assumes that the proportions of men and women *admitted* into public institutions during extensive periods, represent, as, on the whole, they probably do represent, the cases which *occur* for the first time, and remarks as follows: "Having thus shown that in the principal hospitals for the insane in these kingdoms, the proportion of men admitted is nearly always higher, and, in many cases, much higher than that of women; and as we know that the proportion of men in the general population—particularly at those ages when insanity most usually occurs—is decidedly less than that of women, we can have no grounds for doubting that men are actually more liable to disorders of the mind than women." With Dr. Jarvis, we think that any statement in regard to peculiar liability of either sex "must vary with different nations, different periods of the world, and different habits of the people;" and we may add that the peculiar constitution of the American people, as native and foreign born, manufacturing and agricultural, may afford elements for variation, not only in the number of the sane of both sexes, but also in the proportionate relation of the insane.

Leaving this question still in doubtful confusion, we shall examine, under each head, into the influence which sex exerts in the causes and results of mental disorders.

*b. Age at which Insanity first appears.* We are surprised to find such a scarcity of materials upon one of the most important and interesting points in the whole subject of our researches. We have been unable in many of the reports to find the least reference made to this inquiry; but as the information derived from other reports embraces a large number of cases, what we have obtained in this way may be made the basis of our calculations. Without any regard to sex, we find the ages of first appearance of mental disorders to be as follows, in thirteen institutions, bearing in mind always that we can never know certainly, from the accounts of family or friends, the exact time when eccentricity or morbid peculiarities of disposition first exhibited themselves.

*Age at which Insanity first appeared.*

(STATISTICS OF THIRTEEN UNITED STATES INSTITUTIONS.)

| | No. of Cases. | Percentage for each period. |
|---|---|---|
| Under 20 years of age . . . . . | 1724 | 13·8 |
| 20 to 30 " " . . . | 4421 | 35·3 |
| 30 to 40 " " . . . . . | 3117 | 24·9 |
| 40 to 50 " " . . . | 1880 | 15·0 |
| 50 to 60 " " . . . . . | 861 | 6·9 |
| 60 to 70 " " . . . | 354 | 2·8 |
| Over 70 " " . . . . . | 115 | ·9 |
| Total . . . . . . . | 12,472 | |

It will thus be seen that more than 75 per cent. of the insane embraced in this analysis became so between 20 and 50 years of age. We are disposed to think that a larger number become insane between the ages of 20 and 25 than at any other period of five years, but we cannot assert this confidently. A table like that given above, although instructive in some respects, is not perfect, unless we examine into the proportion in the general population of those who are between the ages mentioned. Accepting the census of 1850, with all its imperfections, as our standard, we exhibit in the table the number at each age in the general population; to which the percentage of insane, considered as to the appearance of insanity at corresponding ages, may be added as a means of comparison.

| | General population. | Insane. |
|---|---|---|
| Under 20 years of age . . . . | 52·4 per cent. | 13·8 |
| 20 to 30 " " . . . | 18·4 " | 35·3 |
| 30 to 40 " " . . . . . | 12·1 " | 24·9 |
| 40 to 50 " " . . . | 7·9 " | 15·0 |
| 50 to 60 " " . . . . . | 4·7 " | 6·9 |
| 60 to 70 " " . . . | 2·6 " | 2·8 |
| Over 70 " " . . . . . | 1·5 " | ·9 |

From this comparative table, we learn the interesting fact, which a simple reliance on the previous figures would have left undiscovered, that the ages between 30 and 40 are the most liable to insanity, and that the other periods of life are liable in the following order:—

| | |
|---|---|
| From 30 to 40. | From 60 to 70. |
| " 20 to 30. | Over 70. |
| " 40 to 50. | Under 20. |
| " 50 to 60. | |

Insanity attacks the two sexes at different periods. Thus, of 9951 cases of mental aberration comprised in the preceding statistics, of which 5211 were males and 4740 females, it is found by a comparison which we have made of the prevalence of insanity in either sex at particular ages, with the number of the general population at corresponding ages, that in many cases while one sex predominates in the general population, the other may be in a greater proportion among the insane. We of course assume that the figures taken from our asylum statistics may be taken as a fair standard of the relation of the insane of each sex outside, a mode of reasoning which we admit allows of many objections. We find as the result of these inquiries that—

Males are more liable under 20 years of age.
Females are rather more liable from 20 to 30 years of age.
No difference of liability exists between 30 and 40 years of age.
Females are decidedly more liable between 40 and 50 years of age.

Females are decidedly more liable between 50 and 60 years of age.
Males are decidedly more liable between 60 and 70 years of age.
Males are decidedly more liable over 70 years of age.

If, instead of basing our calculations as to the prevalence of insanity in each sex at particular periods upon the age of first appearance of insanity, we should take the *age of admission* into insane institutions as our guide, we might arrive at slightly different conclusions. But as that mode of classification is not a fair test of the period of life which is most subject to mental disorder, and as the information elicited is more interesting to those who devote themselves to the care of the insane than to the general reader, we shall only briefly allude to the ages at the time of admission, as follows, in statistics of 11,598 cases :—

More patients are admitted between 20 and 30 years of age ; and then in the following order: 30 to 40, 40 to 50, 50 to 60, under 20, 60 to 70, and, lastly, over 70. We find by reference to the statistics of Dr. Earle,* of 1710 admissions into Bloomingdale Asylum, (N. Y.,) that our conclusions coincide exactly with his.

It will be seen that some difference is observed here between the two modes of viewing the question of age; and it may be shown that, as far as the sexes are concerned, men are admitted in larger proportional numbers into the institutions from which we have quoted these statistics, between the ages of 30 and 40, 40 and 50, and 50 and 60 ; and that women occupy a similar pre-eminence at ages under 20, from 20 to 30, 60 to 70, and over 70.

*c. Single or Conjugal Condition.*—It is not our intention here to dwell upon domestic difficulties and ill-assorted matrimonial alliances as causes of mental aberration. However powerful they may be, we must not lose sight of the occasional probability of attacks of insanity occurring, and leading to the very results—painful distrusts and exhibitions of temper in the family circle—which are so often regarded as the causes instead of the effects. We touch lightly here upon such domestic calamities, and refer merely to the actual condition of celibacy, married life, or widowhood, preferring to mention only the fact that the patient was single or married, without inquiring whether he may not have remained single because he had exhibited some mental peculiarity. From the recorded statistics of 20 institutions—and we refer to none other than American institutions whenever we use the term—we have 25,721 cases placed in the category of single, married, widowed, and divorced, which we may classify, as follows :—

* History of the Bloomingdale Asylum for the Insane, p. 65 ; New York, 1848.

| | |
|---|---|
| Single . . . . . . . . | 12,462 or 48·4 per cent. |
| Married . . . . . . . | 11,150 or 43·3 " |
| Widowed . . . . . . . . | 2,092 or 8·1 " |
| Divorced . . . . . . . | 17 |
| | 25,721 |

We should naturally infer that married life would be less frequently a cause of insanity than celibacy, from its being generally a more settled condition of existence, and less liable to fluctuations of agitations and emotions of the mind; and yet the thousand sources of anxiety which a sensitive mind may feel for the happiness of those immediately around him may disturb the mental equilibrium of the married state, and induce morbid changes in his disposition and attachments.

When we consider *the sexes in their relation to the married state*, we find the sex given in 20,281 instances of those mentioned above. These we may arrange as follows:—

*Males:*

| | |
|---|---|
| Single . . . . . . . . . | 5,772 or 55·5 per cent. |
| Married . . . . . . . | 4,090 or 39·3 " |
| Widowers . . . . . . | 537 or 5·1 " |
| | 10,399 |

*Females:*

| | |
|---|---|
| Single . . . . . . . . . | 4,233 or 42·9 per cent. |
| Married . . . . . . . | 4,311 or 43·6 " |
| Widows . . . . . . . . | 1,338 or 13·5 " |
| | 9,882 |

We thus see that a greater proportion of married females to the whole number of insane females exists than of married males to the whole number of insane males, while the single males are in a much greater proportion than the single females. The widows are far more numerous in proportion, according to this estimate, than the widowers. The experience of American institutions on the subject of marriage or celibacy, as a cause of mental aberration may, therefore, be summed up as follows:—

Out of every 1000 cases of insanity, without regard to sex, 433·8 are married, 484·5 single, and 81·3 widowed; and of every 1000 cases of the male sex, 555 are single, 393 married, and 51 widowers; while of the same number of females, 429 are single, 436 married, and 135 widows.

*d. Occupations.*—We can draw no inferences as to the greater prevalence of mental disorder in particular trades or occupations from the mere statement of the fact that a certain number of persons who are engaged in them are insane, unless we also make some estimate of the proportion which occupations bear to each other in the general population. Such an estimate is possible, when we have such facilities for obtaining it as are furnished by the United States census returns. Although errors may exist in it, so also may mistakes creep into the statistics of occupations furnished by ignorant persons to insane institutions. It is, therefore, for ordinary purposes, sufficiently reliable as a means of comparison. For convenience sake, we may adopt a system of classification similar to that suggested originally in the census statistics of Great Britain, and modified so as to adapt it better to the peculiar circumstances of the United States. Any classification must be difficult, but this is perhaps sufficiently simple. We give the percentage of each class in the general population, and of the insane of each class in 7329 cases, collected from the statistics of fourteen American institutions. The majority of our insane hospitals, probably believing the subject a matter of less importance than the minority are disposed to consider it, pass it by entirely without remark of any kind.

*Occupations of the Insane previous to the Attack.**

| | Percentage in gen. pop. | Percentage among Insane. |
|---|---|---|
| 1. Commerce, trade, manufactures, mechanic arts, and mining. | 29·72 | 39·90 |
| 2. Agriculture. | 44·69 | 29·10 |
| 3. Labor, not agricultural. | 18·50 | 13·98 |
| 4. Army and navy. | ·10 | ·70 |
| 5. Sea and river navigation. | 2·17 | 3·50 |
| 6. Law, medicine and divinity. | 1·76 | 7·00 |
| 7. Other pursuits requiring education. | 1·78 | 4·00 |
| 8. Government civil service. | ·46 | ·34 |
| 9. Domestic servants. | ·41 | .54 |
| 10. Other occupations. | ·41 | ·90 |

We thus see a disparity, which may be made much more manifest by an arrangement under separate heads of the principal occupations, which bear a greater ratio to the insane than to the general population, or the reverse. It will be seen, as might naturally be expected, that professional pursuits—and especially the learned professions—are more liable to insanity than those which are not characterized by great mental tension. Farmers, devoted to a quiet life of agricultural occupation, and removed from causes which distract the mind and harass the spirits of the busy commercial world, are able to live more regularly than the par-

* This table, it may be remarked, embraces in the first column the employments of the white and free colored only, while the other *may* (we do not know that it does) include also the slave population. The number of slaves insane is, however, insignificant.

ticipants in active city life, and hence are found low down in the scale of liability to insanity. We should expect, in our political system, with changes going on perpetually, that government officials would rarely be attacked with mental disorder connected in any way with their occupation, because the system of rotation does not often allow them a political life of sufficient duration to disturb their mental equilibrium.

*Occupations which bear a greater ratio to the number of the Insane than to that of the General Population.*

The learned professions—medicine, divinity, and law.
Other pursuits requiring education.
Sea and river navigation.
Commerce, trade, manufactures, mechanic arts, and mining.

*Occupations which bear a greater ratio to the number of the General Population than to that of the Insane.*

Agricultural pursuits.
Government civil service.

It is scarcely worth while inquiring into the relative liability of particular trades, such as shoemaking, tailoring, etc. which are embraced in the main headings given above, although we may devote a moment's consideration to classes 6 and 7 in the previous table, both of which are connected in some way or other with mental development or education. Leaving out of view those illustrations of each which furnish so trifling a number of cases as to make their statistics comparatively worthless, it will be interesting to know how the learned professions, in which we include clergymen, lawyers, doctors, and students generally, compare as to liability to mental aberration; and how also pursuits requiring education, such as those of teachers, artists, druggists, engineers, etc., differ in their tendencies to insanity. The order seems to be as follows:—

| Law, medicine, and divinity. | Pursuits requiring education. |
|---|---|
| Students. | Artists.* |
| Lawyers. | Druggists. |
| Physicians. | Teachers. |
| Dentists. | Musicians. |
| Clergymen. | Engineers, etc. |

Comparing these two classes with one another, we have the educated classes, *par excellence,* liable to insanity in the following order:—

* We do not doubt that we might assign to *authors* one of the highest places in the table, if we felt ourselves justified in using the meagre statistics we have at hand in regard to them.

| | |
|---|---|
| Artists. | Physicians. |
| Druggists. | Dentists. |
| Students. | Clergymen. |
| Teachers. | Musicians. |
| Lawyers. | Engineers. |

No statistics have been furnished in the United States census returns of the number of females occupied in the different branches of industry, so that it would be impossible for us to make any comparative table of occupations of that sex, as predisposing to insanity. Domestics, seamstresses, and teachers seem to be in large numbers, and so also do the wives and daughters of farmers and laborers, whose occupations must be of a domestic nature; but all these classes are numerous also in the general female population, and we should therefore expect insanity to be prevalent among a large number in each.

*e. Hereditary predisposition* doubtless exists in a far greater number of cases than is generally supposed. Few of our institutions, however, class the inheritance of mental alienation among the causes in their lists, and in only one or two instances is any mention made of it at all. We know from other sources of information, such as those supplied to us by European channels, that particular forms of insanity may be transmitted from parent to child, and that the eccentricities and disordered understanding peculiar to the one may, by hereditary predisposition, appear in all their force in the mental development and characteristics of the offspring. So much did Esquirol believe in the transmission of hereditary insanity that he considered that six-sevenths of all his patients had been blighted by such a heritage. Believing that this predisposing cause of insanity is so imperfectly recognized, or, if recognized, so unsatisfactorily and unreliably recorded, that it assumes statistically but little importance in the reports of hospitals for the insane, it is deemed expedient to extract from Dr. Earle's "History of the Bloomingdale Asylum" (p. 80) a few remarks on hereditary predisposition in cases admitted into that institution. "Of 1841 patients, 323—of whom 187 were males, and 136 females—are recorded as having one relative or more insane; this is equivalent to $17\frac{1}{2}$ per cent. The percentage in each sex, taken separately, is as follows: men, 17·16; women, 18·11. It is not to be presumed, however, that this is even a near approximation to the number actually having relatives of disordered mental powers.

"*Of the men* included in the foregoing table, 118 inherited the predisposition from direct ancestors, and 33 of these had other relatives insane. Of the remainder, 68 had collateral relatives insane, but no direct ancestors; and one had a child insane. Of the 52 who had insane parents, it was the father in 27 cases, and the mother in 25. In one of these, both father and mother had been deranged. It is also stated that two of those included under the term hereditary, had ancestors, both paternal and maternal, who were subject to the malady. *Of the women*, the predisposition was transmitted from direct ancestors in 89, of whom 67 had other relatives insane. In the remaining 42, the disease is stated to have appeared only in persons collaterally connected; and in 5 cases in their children alone. There are 18 cases in which it is mentioned that the father was insane. In 1 case, the father and mother were both deranged. In the case where it is asserted that the whole family were insane, it is said that all her father's family, which consisted of 12 children, had been deranged, and that their insanity did not, in a single instance, make its appearance before the age of 21 years. Two brothers were patients here in 7 instances, 3 brothers in 2, a brother and sister in 2, 2 sisters in 3, 2 sisters and 2 of their cousins in 1, mother and son in 3, father and son in 1, father, daughter, and her son in 1, mother and daughter in 3, and uncle and niece in 1. It is obvious that the foregoing statistics are not sufficiently full or definite to be adopted as accurate data from which to estimate the proportion of the insane in whom it is transmitted from the father's or the mother's side, or any of the other important questions involved in the subject."

Such are the main points of interest in relation to the subject of family predisposition; they embrace much more minute details than can be found elsewhere in American reports. We regret our inability to make them more copious. It is gênerally supposed that those cases of insanity which are transmitted in families do not respond to treatment as readily as others which are spontaneous in their origin.

*b*. While referring to the hereditary transmission of insanity, we may appropriately speak of the influence of *marriages of consanguinity* in the production of mental alienation. The vital statistics of marriage are not dwelt upon in any of the statistical records which it has been our province to consult. Dr. Bemiss,* of Louisville, Ky., has made quite extensive investigations into the claims of intermarriage of blood-relations, to a place among the predisposing causes of disease in the offspring, to which we may refer those who are desirous of studying the subject more fully. Insanity results far less frequently than forms of imperfect development, such as give rise to idiocy, deaf-muteism, etc. The State Government of Ohio seems to have taken considerable trouble to collect details connected with this point; and although the report upon it is

* Report on the Influence of Marriages of Consanguinity upon Offspring, by S. M. Bemiss, M.D., Louisville, Ky. Transactions of the American Medical Association, vol. xi. p. 319. 1858.

very imperfect, or totally deficient in some counties, Dr. Bemiss has been able to collect materials enough on which to base the following calculations: "If the same ratio be supposed to exist throughout the Union, (as in the Ohio Report,) there would be found, to the twenty millions of white inhabitants, six thousand three hundred and twenty-one marriages of cousins, giving birth to 3909 deaf and dumb, blind, idiotic, and insane children, distributed as follows:—

| | |
|---|---|
| Deaf and dumb | 1116 |
| Blind | 648 |
| Idiotic | 1854 |
| Insane | 299 |

Then, if the figures of the last United States census still applied to our population, there would now be found in the Union—

9,136 deaf and dumb, of whom 1116 or 12·8 per cent. are children of cousins.
7,978 blind, of whom 648 or 8·1 per cent. are children of cousins.
14,257 idiotic, of whom 1844 or 12·93 per cent. are children of cousins.
14,972 insane, of whom 299 or 1·9 per cent. are children of cousins."

Such are the calculations of probabilities founded upon the history of a large number of illustrations of infirmity in the United States, which have been connected in some way—we will not say positively dependent on—circumstances of relationship such as we have described. They form an interesting supplementary matter for investigation, after the remarks already made on hereditary predisposition.

*g. Education, systems of religion*, etc., doubtless exert a powerful influence upon the mental development of the individual; but no accurate data are obtainable by which we may recognize how extensive that influence may be in a country like our own, so differently constituted from every other in its systems of education and its numerous forms of religious belief.

Education, when carried so far as to produce overstrained mental exertion, will frequently, doubtless, in the more delicately constituted organization of some children, prove occasionally mentally injurious, and sow the seeds of future perversion of the intellectual and moral faculties. How far this influence is exerted in this country, we are unable to say.

### 3. HISTORY OF THE ATTACK.

Having studied the personal history of the patient previous to the attack, we have next to inquire into the statistics of the most interesting points connected with the history of the attack itself. Although many of the causes which excite to insanity might be said properly to belong to the general field of inquiry through which we have already traveled, yet the causes and results may be more appropriately studied in conjunction than in any other mode. Great difficulty must exist sometimes in defining the exact lines, where one subject begins to be perfectly distinct from another, and hence any method of classification must have its defects. We shall find, indeed, that the two divisions we have adopted will occasionally run into each other, as when we refer to the influence of sex or marriage, both of which belong to the personal history of the patient, upon mortality or recovery, subjects appropriately considered in relation to the history of the attack. It seems advisable, however, to adopt some classification of this kind, as a means of avoiding the confusion which would result from the clustering together of so many points of interest in a chaotic mass.

Included in this department of our subject are the exciting causes of insanity, whether moral or physical; the statistics of recovery and mortality; and the influence of sex, marriage, and other conditions upon each. The recorded statistics on some of these points are copiously illustrative, but many of the reports of institutions neglect them entirely for the sake of dwelling upon other matters of purely local interest.

*a. Causes of Insanity.* The Tables of Causes which are furnished must always be very imperfect. Many of them are imaginary, and were two sane members of the same family consulted as to the antecedents of the attack, it is exceedingly doubtful whether they would assign the same causes. Especially is this the case in hereditary insanity, to mention which is by some regarded as a slur upon the family escutcheon, the publication of a stain upon family good name, by those who have no right to intrude within the privacies of a home-circle. We have heard of cases in which the father of a family had positively denied the existence of hereditary insanity in the presence of a relative, who was more honest, and less disposed to embarrass the researches of the physician. Against all these sources of error, the incompetency of deciding as to the true cause, the unwillingness to expose family infirmities, the conjectural idea that some one cause may have been more potent than another, and the concealment of domestic troubles and difficulties, the physician has to contend in his attempts to study the subject intimately.

The effect is doubtless very often made to supply the place of the cause, and because a patient exhibits a tendency to certain forms of mental alienation, the form itself is assigned as the cause of the malady. For example, when we find, as we very often do, religion placed among the causes of insanity, on what strict premises can we infer that anxiety in regard to a future state has been the true moral cause? Insanity may have existed unsuspected, and when fully developed, may have assumed the form of religious mania; but surely a thousand causes might have existed previously. The fallacies of the tables which occupy a large space in some of our reports are numerous, and yet the greatest refinement of divisions, down to the most trivial of probabilities, is practiced without any attempt at a classification which would throw light upon the often obscure etiology of the affection. Added, therefore, to the ignorance or concealment of friends of the patient, we have often the confusion of subjects and the deficiency of classification of those who have the ability to simplify. Between the two dilemmas, one is almost tempted to make the simplest division of causes; for instance, into those which produce insanity from the mere brooding over imaginary or real griefs, and of those which operate otherwise; in some general plan of this kind disposing thus of the whole subject. With the materials before us, however, we must extract, if possible, as much really useful statistical information as is practicable under the circumstances, even though the causes stated are often mistaken ones.

The general division of causes, which has been usually adopted, is into those which are *predisposing*, and those which are *exciting* or *productive* of insanity. Numerous circumstances of constitutional predisposition, of temperament, education, sex, and age, exist to induce a predisposition to insanity; while who can estimate the number of exciting causes enumerated as the active agents in its production?

*Exciting Causes, Moral and Physical.* We may again subdivide the exciting causes into moral and physical; the latter class, it has been asserted, is more frequently met with among the lower ranks of society, while the former belongs to a higher class, whose intellects are more developed, and whose minds are subjected to more extensive influences. The passions and emotions, when viewed as causes of mental alienation, are referred to the class of moral causes. Physical causes include such agents as act by some influence exerted externally, and only secondarily affect the nervous system, while moral causes exert a decided influence on it from the very inception.

We have taken considerable care and trouble in classifying the moral and physical causes of insanity in 11,259 cases, furnished in the reports of a large number of institutions. With a mental reservation, that accuracy is not always perfectly attainable, we may gain a certain amount of intelligence of the early history of the attack. We may illustrate what we mean by moral and physical causes in the following examples:—

*Moral causes*, as domestic difficulties, religious anxiety, political excitement, intense mental application, etc. *Physical causes:* Intemperance, ill health, epilepsy, sensuality, etc.

The distinction is generally an obvious one, and the cases are readily arranged under one or the other heading. It will be seen by the following table that the physical causes predominate over the moral—a fact which is denied as probable, when applied to many of the European institutions, but yet which has been previously recognized as true in regard to the statistics of our own insane hospitals:—*

*Table of Moral and Physical Causes of Insanity,* (11,259 *cases.*)

| Moral Causes. | | Physical Causes. | | |
|---|---|---|---|---|
| Domestic troubles and griefs | 928 | Ill-health and unclassified diseases | 2,388 | 3067 |
| Religious anxiety | 792 | Fevers | 199 | |
| Mental anxiety | 721 | Epilepsy | 319 | |
| Financial difficulties, reverses of fortune, etc. | 652 | Cerebral disease | 117 | |
| Loss of friends | 585 | Paralysis | 44 | |
| Disappointment in love, ambition, etc. | 576 | Intemperance and dissipation | | 1202 |
| Excessive study or application to business | 165 | Conditions peculiar to women | | 891 |
| Fear and fright | 126 | Vicious habits and indulgences | | 514 |
| Defective education | 37 | Wounds and blows | | 250 |
| Uncontrollable temper | 32 | Excessive use of opium, tobacco, etc. | | 129 |
| Nostalgia | 29 | Exposure and loss of sleep | | 123 |
| Political excitement | 22 | Spiritualism | | 94 |
| Unclassified moral causes | 40 | Exposure to sun or heat | | 74 |
| | | Over-exertion | | 62 |
| | | Old age | | 32 |
| | | Unclassified physical causes | | 172 |
| Total | 4,649 | Total | | 6,610 |

One great difficulty in the perfect isolation of the physical causes is the condition, assigned most frequently as a cause, which, meaning nothing or everything according to the interpretation of the person who furnished it, is termed "Ill health." Whether it should be called a cause or an effect must often depend on circumstances of the history of the case which are beyond our powers of research to discover. But we place it where it generally has a position, among the physical causes of insanity, although it may sometimes include and conceal others, such as intemperance and dissipation, or sensuality. We cannot point out here the great defects of any such system of classification; we refer now merely to the fact that the moral causes constitute only two-fifths, and the physical causes three-fifths of the whole number of causes above given. A glance at the table shows the order in which causes, taken as a whole, and not as divided into moral and physical, are arranged as exciting to insanity, and exhibits that ill health and intemperance rank first, domestic troubles and griefs next in order, then the conditions peculiar to women, and so on.

* Bucknill and Tuke, Manual of Psychological Medicine, Philadelphia, 1858, p. 257.

*Sex* has an important influence in the distribution of moral and physical causes. The circumstances of exposure form so decided an element in the latter class, that we would naturally suppose man to be much more liable to them than woman; while the latter would suffer more from domestic troubles, and from loss of those cherished by her, from the fact that, in the absence of occupation, she would probably brood over her misfortunes. Thus, of 3118 moral causes included in our table, in which the sex was known, 1585, or 51 per cent., were females, and 1533, or 49 per cent., males. If we omit, for a moment, from the list of causes of insanity, financial difficulties, politics, and application to business, which are almost exclusively sources of insanity of males, we shall find, in the more delicate emotions and passions, that woman becomes insane from moral causes in 57 cases out of every hundred, while man only suffers in 43 cases.

The reverse is true of physical causes, and *a fortiori*, if we exclude from consideration diseases peculiar to females. Including all the causes of this class in both sexes, the males are to the females as 53 to 47; excluding the diseases of women, a proportion exists of 66 males to 34 females.

Having thus accounted for the production of the attack of mental aberration, we may watch its progress until it becomes introduced to the care and attention of an institution devoted to its protection and relief. Several considerations are worthy of being studied from the time of the invasion of the disease up to its actual admission to an insane hospital. By that time it will have assumed a definite form, such as dementia, melancholia, etc., and have possessed some interest in its duration; its probable prognosis for recovery, or the reverse, being often influenced by the fact of its being an acute or a chronic case when admitted.

*b. The Special Forms of Insanity.* It is not our province to suggest any system of classification different from those adopted by experienced authorities on this subject, and we therefore, for simplicity's sake, employ that followed in the Report of the Pennsylvania Hospital for the Insane,* embracing the division into mania, melancholia, monomania, dementia, and delirium, the last of which, being so very unfrequent, is scarcely worthy of being referred to here. In the words of the Report of the Bloomingdale Asylum: "The nosology of mental diseases is still so imperfect, that it is difficult to make an arrangement of cases which would be of any material value, either practical or theoretical. Indeed, there are scarcely two physicians who would classify a series of cases, such as are admitted

---

* Report of the Pennsylvania Hospital for the Insane for the year 1859, p. 45. By Thomas S. Kirkbride, M.D. 1860.

into any institution, in precisely the same manner. A case called partial insanity by one person might be termed monomania by another. That which one records as monomania, another would place under the head of melancholia. There being no definite line between mania and dementia, a given case might be placed under the former by one physician, and under the latter by another. A perfect nomenclature of insanity is a great desideratum."*

Now that the terms are rather more intelligible than they seem to have been formerly, the degree of confusion is less marked, and we may arrange under a few heads almost all of the forms which insanity assumes. In 7322 cases, embracing the four forms, mania, melancholia, monomania, and dementia, the number of each is as follows :—

| | | |
|---|---|---|
| Mania | 3789, or 51·7 | per cent. |
| Melancholia | 1366, or 18·7 | " |
| Dementia | 1265, or 17·3 | " |
| Monomania | 902, or 12·3 | " |

The influence of *sex* is visible in the distribution of the special forms of insanity, in the same number of cases, including 4230 males and 3407 females, as follows :—

| | | | | |
|---|---|---|---|---|
| Of the males, | 2090, or 51·7 | per cent., | were attacked with | mania. |
| | 781, or 19·3 | " | " | dementia. |
| | 648, or 16·0 | " | " | melancholia. |
| | 520, or 12·9 | " | " | monomania. |
| Of the females, | 1699, or 51·7 | per cent., | were attacked with | mania. |
| | 718, or 21·8 | " | " | melancholia. |
| | 484, or 14·7 | " | " | dementia. |
| | 382, or 11·6 | " | " | monomania. |

While each sex, therefore, is attacked with mania in an equal proportion, men are more often the subjects of dementia than women, and the latter more often suffer from melancholia. This fact bears a decided influence upon the curability of the sexes, as we shall hereafter see.

We need not enter into a minute consideration of the various subdivisions of each form of insanity, such as that species of mania which is connected with the puerperal state, or that which assumes incendiary, homicidal, or suicidal tendencies. Interesting as they would be, viewed as collateral subjects of inquiry, it is scarcely appropriate that we should attempt any brief analysis of so much that may be found ably discussed in special treatises on these various forms of mental alienation. Puerperal insanity has been very fully investigated by numerous medical writers, and

* History, etc. of the Bloomingdale Asylum, New York, 1848.

has lately undergone a careful statistical analysis in the pages of the *American Journal of Insanity.**

*c.* The *influence of season upon the admissions* into institutions for the insane is sensibly apparent for both sexes combined, and for each sex separately. Thus we find in 21,072 cases the following results:—

*Influence of Season upon Admissions.*

| | | No. of Admissions. | Per cent. | |
|---|---|---|---|---|
| Winter months. | December | 1469 | 6·9 | 20·6 per cent. |
| | January | 1513 | 7·1 | |
| | February | 1375 | 6·5 | |
| Spring months. | March | 1665 | 7·9 | 26·6 per cent. |
| | April | 1845 | 8·7 | |
| | May | 2109 | 10·0 | |
| Summer months. | June | 2293 | 10·8 | 29·2 per cent. |
| | July | 2063 | 9·7 | |
| | August | 1809 | 8·5 | |
| Autumn months. | September | 1755 | 8·3 | 23·4 per cent. |
| | October | 1669 | 7·9 | |
| | November | 1507 | 7·1 | |

It is thus exhibited that the summer months are those in which the greatest number of insane patients are admitted into our institutions. June seems to take the precedence, a fact which coincides with the opinion of M. Esquirol, and of MM. Aubenel and Thore,† as the result of their European investigations; but May ranks higher in the list of American monthly admissions than they are disposed to place it. The order of the seasons, derived from the above table, is summer, spring, autumn, and winter. The admissions do not of course represent the *occurring* cases of insanity, but only such as are admitted into institutions; some of them being chronic cases, while many of them are acute. The results for the sexes, individually, are as follows:—

| | Per cent. Winter. | Per cent. Spring. | Per cent. Summer. | Per cent. Autumn. |
|---|---|---|---|---|
| Men | 21·0 | 26·1 | 29·1 | 23·6 |
| Women | 19·16 | 27·0 | 29·7 | 23·5 |

The relative frequency of admission being very nearly the same in each sex during the summer and autumn, it will be seen that a greater proportion of the men are admitted during the winter, and of the women during the spring. It will be shown hereafter that season also influences the termination of insanity in recovery or death.

* Observations upon Puerperal Insanity, by Richard Grundry, M.D., Assistant Physician to the Southern Ohio Lunatic Asylum. (Reprint). Utica (N. Y.) State Lunatic Asylum, 1860.

† Bucknill and Tuke, op. cit. p. 249.

*d. Duration of Insanity previous to Admission.*—The details of 10,304 cases furnish the fact that 60 per cent. of the cases admitted into our American institutions have only been insane for a few months, the greater majority being less than 6, and a few ranging from 6 to 12 months; while more than a quarter of the cases, about 2600 in all, have been insane from 1 to 5 years. The result of such cases must, of course, vary according to the general duration of the attack, chronic cases being much more intractable than acute. But this branch of our inquiry belongs more appropriately to the consideration of the terminations of insanity.

### III. INSANITY CONSIDERED IN ITS RESULTS.

The experience of insane institutions throughout the world exhibits great diversity in the results of different modes of treatment, in the restoration to health or in the tendency to relapse or death. So much depends upon other circumstances, too, than the mere routine of treatment, and so many more influences operate to produce a change in the condition of a patient in some parts of the country than in others, that the measures adopted for his restoration may prove less beneficial in one institution than in another. Climate is not one of the least important of these influences, and season exerts a decided effect upon the curability or non-curability of insanity. We need but consider three terminations of an attack of insanity, viz., restoration to good health, confirmed dementia, and death. And yet how difficult to decide in regard to a perfect restoration! Must we merely take the statistics as we find them, and consider the case cured, because at the time of departure from an institution it was regarded as "cured" or "recovered?" How much more perfect would be a history of the after-life of the individual during the first year, or two years, afterwards, for instance, giving information of the permanency of the cure or the reverse! It is a fact that can scarcely admit of contradiction, that the friends of a patient who has been discharged "cured" from an institution, will, occasionally, when a relapse has occurred, have diminished confidence in the mode of treatment, and decline to send him back to that institution, or perhaps place him under the charge of some other. These are the cases which prevent statistics from being perfectly accurate; the early history is incomplete, and his friends may, perhaps, conceal the fact of his previous residence in another institution. As this, however, may apply to all such cases and all such establishments, a certain amount of practical information may be derived from the statistical records of recoveries, relapses, and mortality. Death alone is certain; its statistics are invariably fixed; but the history of the progress of the case toward a fatal termination assumes a thousand varied phases.

It has been frequently asserted that insanity is a morbid condition upon which remedies may be employed in vain; that incurability is the rule, and recovery the exception. We need not appeal to European sources for a refutation of this fallacy; our own institutions afford abundant means of exhibiting how frequently the careful attention and skill of the physician are rewarded and his labors blessed with abundant sources of congratulation. Very few, however, have it in their power to watch the cases which leave them, improved, or removed sometimes too early, doubtless, by the over-anxious interference of friends. Those who die while under the care of institutions afford only an approximative means of calculating the proportionate mortality; for we cannot tell how many may have left the institution to die at home. Another difficulty in the way of accuracy of details exists in the admixture of chronic cases, which present but slight prospects of recovery, with those acute or less chronic cases, of which hopeful anticipations may be indulged. The proper mode to estimate the proportionate number of recoveries and deaths is to compare them, wherever it is practicable, with the whole number of admissions; but the great majority of the institutions follow a different method, and base their calculations upon the discharges, instead of the admissions. We shall adopt that mode here, and at the same time, if practicable, compare the result with the number of admissions also.

1. *Recovery* must depend upon various circumstances of age, sex, season, etc. Restoration is the result in a very large nnmber of cases, but the chances for a favorable result must be influenced largely by conditions that operate in all diseases, whether mental or physical. A general idea of the proportion of recoveries may be given, however, without specifying what were the extraneous influences at work to modify the nature of the termination of the attack. In 15,235 cases discharged from a number of American hospitals, the recoveries were 6549, or 42·9 per cent. of the whole number; while in 58,607 cases admitted into 33 hospitals, the number of recoveries was 24,937, or 42·5 per cent. It seems to make no material difference whether we calculate recoveries according to the discharges or the admissions, the percentage being very nearly the same. This percentage is higher than that given many years ago by Esquirol, as the result of the experience of the best English and French hospitals for the insane, the ratio of recoveries to admissions being at that time only 39 per cent. in nearly 22,000 cases.

*a.* The *sex* of 10,679 cases admitted into a number of hospitals, and examined in their proportion of restorations, gives the following ratio:—

| | | | | |
|---|---|---|---|---|
| 2452 | recoveries in | 5699 | male admissions, or | 43·0 per cent. |
| 2230 | " " | 4980 | female " " | 44·8 " |
| 4682 | | 10,679 | | 43·8 per cent. |

In 17,833 cases discharged from 23 institutions, the proportion of recoveries in each sex is as follows:—

| | | | | |
|---|---|---|---|---|
| 4095 recoveries in | 9200 males discharged, | or | 44·5 | per cent. |
| 3947 " " | 8633 females " | " | 45·7 | " |
| 8042 | 17,833 | | 45·0 | per cent. |

Recovery is, therefore, more probable among females than among males, a fact which has been often noticed by those interested in insane matters. This more favorable result in that sex depends on the form of the attack, etc., and sometimes on revolutions in her system, which produce happy changes when the resources of medical art have been ineffectual. Women suffer much more from melancholia than men, while the latter are more subject to dementia. The latter being in the majority of cases incurable, as we shall presently see, some reason seems to exist in this fact why the female sex should recover more often from insanity than the male.

*b. Season*, too, slightly influences the period of recovery, but our statistics are too meagre for perfect reliance on any deductions. In the Pennsylvania Hospital for the Insane, the recoveries have been as follows:—

| | |
|---|---|
| Winter months | 360 |
| Spring " | 410 |
| Summer " | 475 |
| Autumn " | 411 |

But we have no satisfactory record of the experience of other institutions.

*c.* The *form* assumed by the attack modifies the prognosis as to the result. Thus in 6306 illustrations of four forms of insanity, discharged,—

3576 were cases of mania, of which 2620, or 73·2 per cent., had recovered.
1400 were cases of monomania, of which 829, or 59·2 per cent., had recovered.
819 were cases of melancholia, of which 457, or 55·8 per cent., had recovered.
511 were cases of dementia, of which 85, or 16·6 per cent., had recovered.

Mania is the form most curable, and dementia that which is least tractable to remedial or other influences.

*d.* The *duration of the attack*, of course, has much to do with the prospects of recovery; the acute cases being much more susceptible of relief than the chronic. Thus of 619 cases that were chronic when admitted into one of our institutions,* only 130, or 21 per cent., recovered; while of 1134 that were recent, 688, or nearly 61 per cent., recovered. In another,† only 20·03 per cent. of cases that were of more than twelve

* Indiana Hospital for the Insane, 1859.

† Report of the W. Virginia Asylum, 1859.

months duration, when admitted, recovered; while 67·08 per cent. of those of twelve months duration or less, when admitted, were restored to health. In one institution,* 80 per cent. of the recoveries during the year ending October, 1859, had taken place "in cases which had been less than three months insane; 87 per cent. in cases which had been less than six months insane; and 93 per cent. in cases which had been less than one year insane. At the same time it should be remembered that in certain exceptional cases recovery may take place after the lapse of many years." What volumes does this not speak for the early introduction of insane patients to the care and attention of insane institutions! Such has been the experience of every one who has devoted any attention to mental pathology. "If," says Dr. Jarvis, "insane persons are allowed to enjoy the means of healing in the early stages of their disorder, about 75 to 90 per cent. can be restored to health." Of 880 cases discharged from five institutions in one year, 726, or 82 per cent., were recent cases.

2. *Mortality of the Insane.* We can readily understand how life may be shortened by attacks of insanity which exhaust the vital forces and so seriously disturb the various functions. An eminent writer assigns, as one of the modes in which the insane may fatally terminate their attacks of insanity, the masking or concealing of dangerous affections by the mental disease, complaints of the patient being frequently overlooked and taken for delusions, and the true pathology not being detected until after death.† Undoubtedly this is frequently true, but whatever be the cause of death, there are certain points which can only be studied in relation to the general subject of mortality, independent of the cause, whatever it may be, that may have terminated life—such as the relative number of deaths to the number of admissions or discharges, and the influence of sex, etc., on mortality.

In thirty-three of the U. S. institutions, the number of deaths based upon 56,405 admissions was 8638, or 15·3 per cent.; while in 15,235 cases discharged from twenty-one hospitals for the insane, 3256 died, or 21·3 per cent. One reason of this disparity between the admissions and the discharges is obvious—a large number of those cases which have undergone no improvement remain in an asylum, and do not, therefore, swell the list of discharges. Compared with the recoveries, we have the following favorable or fatal results in a corresponding number of cases:—

| | Per cent. recovered. | Per cent. died. |
|---|---|---|
| Of those admitted . . . . . . . . | 42·5 | 15·3 |
| " " discharged . . . . . . . . | 42·9 | 21·3 |

*a.* The *sex* is given in 3557 deaths, based upon 18,594 admissions, as follows:—

* Sixth Annual Report of the State Lunatic Hospital at Taunton, Massachusetts, 1859, p. 28.

† Dr. Copland. Dictionary of Practical Medicine. Vol. II. London, 1858, p. 472.

1957 deaths in 9760 male admissions, or 20 per cent.
1600 deaths in 8834 female admissions or 18·1 per cent.

If we study the fatal results in proportion to the discharges of each sex, we have 11,857 cases on which to found an estimate, 2631 of which proved fatal:—

1414 males died, or 22·5 per cent. of the males discharged.
1217 females died, or 21·7 per cent. of the females discharged.

In each mode of viewing the question of mortality of the sexes, we find the males dying in a larger proportion than the females.

*b.* The *seasons* at which the mortality appears to be the greatest are summer and autumn, and afterwards spring and winter. But in a country like our own, which has so many different climates, the effect of season must vary materially according to the locality of the institution, and we are not therefore justified in drawing inferences for the whole country, from the greater tendency which seems to exist toward fatal terminations of insanity in any set of institutions situated in the North or the South. Conclusions arrived at from such premises are not worthy of being recorded.

*c.* We have but little information as to the fatal results of any of the *forms* of mental disorder; the experience of our own institution gives the following mortality of each:—

| | | | | | | |
|---|---|---|---|---|---|---|
| Mania, | 156 deaths in | 1569 | cases admitted, | or | 9·9 | per cent. |
| Melancholia, | 72 " | 819 | " | " | 8·7 | " |
| Monomania, | 21 " | 411 | " | " | 5·1 | " |
| Dementia, | 105 " | 296 | " | " | 35·4 | " |
| Delirium, | 9 " | 11 | " | " | 81·8 | " |

We need not here dilate upon the pathology of those forms of mental alienation which are so violent or so destructive in their nature as to afford but a slight chance of recovery, nor refer more particularly to the greater curability of certain other forms, in which death is the great exception.

*d.* The *causes of death* among the insane are of course very numerous, being modified, however, according to location and season, according to influences similar to those which operate in the general population. Thus, at times when cholera was prevalent, the mortality of some of our institutions, especially those intended for the reception of pauper patients, was materially increased. This will partially account for the greater number of cases in which death was ascribed to diseases of the digestive apparatus in the following statistics. Although so prominent in the list, we are disposed to assign it a lower relative position from the influence which this disease may have exerted. Of nearly 2100 causes of death, gleaned from the records of our asylums for a series of years,

677, or nearly five-sixteenths, were affections of the nervous system, not including exhaustion, which would add a sixteenth more to that class of causes; more than two-eighths (613 cases) were diseases of the digestive apparatus, and the same proportionate number (604 cases) were morbid conditions of the respiratory apparatus, while fevers, accidents, suicides, etc. made up the balance. This result coincides pretty closely with that obtained by Esquirol in the post-mortem examination of more than 600 cases of insanity. He found that three-eighths had died of diseases of the abdomen, two-eighths of diseases of the chest, and three-eighths of alterations of the brain and membranes. Dr. Copland, in calling attention to these investigations of the distinguished psychologist, observes, "the proportion here assigned to the first class of diseases is probably too high, and especially in respect of this country,"* (England.) The remark is applicable also to the mortuary statistics of the insane in the United States.

3. *History of the Recurrence of Insanity.* We have been baffled in a great measure in an attempt to discover what proportion of cases in our hospitals for the insane were not first attacks, but mere recurrences, probably after intervals of apparent sanity in many of the cases, by the little attention paid to this branch of inquiry. It is exceedingly difficult, also, to trace the history of a patient who leaves one institution, after what seemed to be a perfect restoration to health, to enter another, in which the fact of previous attacks may be concealed or not carefully inquired into. Nor does the mere fact stated, that the patient is suffering from a first attack when admitted, prove that he may not have been intermittently or remittently insane or incurable for a series of years. But taking the classification as we find it in the records of three extensive institutions, we have 5370 admissions into institutions classified in the order of attack, as follows :—

*Number of the Attack.*

| | Number. | Percentage. |
|---|---|---|
| First attack . . . . . . . | 3790 | 70·5 |
| Second attack . . . . . . | 924 | 17·2 |
| Third attack . . . . . . . | 309 | 5·7 |
| Fourth attack . . . . . . | 145 | 2·7 |
| Fifth attack . . . . . . . | 75 | 1·4 |
| Sixth attack . . . . . . | 47 | ·9 |
| Seventh attack . . . . . . | 30 | ·5 |
| Eighth attack . . . . . . | 12 | ·2 |
| Ninth attack . . . . . . . | 38 | ·7 |
| | 5370 | |

* Copland, op. cit. Vol. II., p. 473.

Thus, 1580, or 29·4 per cent. of the cases were other than first attacks. About 28 per cent. of the males were second and subsequent attacks, but the percentage of females was somewhat greater, being as much as 31 per cent. But all these statistics are liable to errors and difficulties such as we have already indicated, so that we cannot rely confidently on the accuracy of deductions from them. We need not here inquire into the number of those who are admitted more than once into the same hospital in a series of years, as our information is indefinite and inconsequential.

Such are the prominent topics which we have deemed worthy of investigation, in the recorded statistics of our American institutions for the insane. The omission of several important subjects, which would have added fullness to our remarks, without increasing their value as results of the observations and experience of hospitals in this country, has afforded us an opportunity of studying much more carefully points of general interest, which are not matters of medical curiosity alone. It has not been our object to dilate on abstract questions of insanity, or to point out peculiarities in systems of treatment, or to devote any attention whatever to such considerations as can have attraction to psychologists only. Even some of the minor points of statistical interest have been passed over without remark, mainly because it has not been thought expedient to deduce general laws from meagre results, which have only been recorded in one or two institutions. A complete treatise on insanity would also include all the complications of morbid phenomena which attend or are consequent upon attacks of mental disease, as well as a more full investigation of the post-mortem appearances observed in such cases. The phrenologist may interest himself in the form of the osseous prominences of the insane; the pathologist may watch the progress of the case from its inception to its close, and eagerly pursue his inquiries into the precise portion of the cerebral organs which has undergone a serious lesion, and hope to throw new light where all is darkness; and the therapeutist may strive in vain to procure the recovery of his patient while he allows him to remain exposed to the causes of his malady. It has been the purpose of this article to watch merely the results of the observations of each, and, discarding some of their conclusions as unreasonable and unreliable, to furnish some of those which exhibit most satisfactorily, although occasionally but imperfectly, what the United States has been able to accomplish for the unfortunate sufferers from mental disease who have been the recipients of her active sympathy and philanthropy.

